# H2-antagonist in IgE-mediated type I hypersensitivity reactions: what literature says so far?

**DOI:** 10.1186/s12948-021-00143-y

**Published:** 2021-04-13

**Authors:** Matteo Borro, Simone Negrini, Andrew Long, Sharon Chinthrajah, Giuseppe Murdaca

**Affiliations:** 1 Clinical Immunology Unit, Department of Internal Medicine, University of Genoa and Ospedale Policlinico San Martino, 16132 Genoa, Italy; 2 Sean N. Parker Center for Allergy and Asthma Research at Stanford University, Stanford University, Grant Building, S093, 300 Pasteur Dr., Stanford, CA 94305-5101 USA; 3 Department of Internal Medicine, San Paolo Hospital, Via Genova 30 -, 17100, Savona, Italy

**Keywords:** H_2_-receptor antagonist, Histamine, Type-I hypersensitivity reaction, Allergy

## Abstract

Histamine is a monoamine synthesized from the amino acid histidine that is well-known for its role in IgE-mediated anaphylaxis but has shown pleiotropic effects on the immune system, especially in order to promote inflammatory responses. H_1_-receptor antagonist are common drugs used in mild/moderate allergic reactions whereas H_2_-receptor antagonist are commonly administered in gastric ulcer but showed some properties in allergy too. The EAACI guidelines for diagnosis and treatment of anaphylactic reactions recommend their use as third-line therapy in adjunct to H_1_-antagonists. The purpose of this article is to produce a complete summary of findings and evidence known so far about the usefulness of H_2_-receptor antagonist in allergic reactons.

To the editor,

According to EAACI guidelines for diagnosis and treatment of anaphylactic reactions [1], H_2_ anti-histamines are recommended as third-line therapy in adjunct to H_1_-antagonists. A recent systematic review evaluating acute and long-term management options for anaphylaxis concluded that this combination may require additional research prior to being included in recommended guidelines [2]. Histamine is a well-known immunomodulatory molecule that is able to influence the Th1/Th2 balance through a number of mechanisms and receptors [3, 4]. Among CD4+ T lymphocytes, Th1 cells are known to express higher levels of H_1_-receptor in contrast to Th2 cells that express a significantly greater proportion of H_2_-receptors. Through the binding of H1-receptor, histamine is able to activate a Th1-characterized response by increasing the release of interferon­γ. Alternatively, through H_2_-receptor, histamine is able to suppress a Th1 and/or Th2 response [5] by inhibition of IL-2, IL-4, IL-13, and interferon­γ production [6]. Within dendritic cells, histamine can drive an increase in expression of both MCH-II and the co-stimulatory molecules CD80 and CD86. Binding at the H_2_ receptor on both dendritic cells and monocytes, it can induce the production of IL-10, and, specific to monocytes, downregulate the release of IL-12, thus shifting the Th1/Th2 balance toward Th2-dominance [7]. Within eosinophilic granulocytes, histamine presents contrasting effects: low concentrations induce an increase in chemotaxis through H_4_-receptors, whereas greater concentrations are responsible for decreased chemotaxis via H_2_-receptors [6].

Despite their use in IgE-mediated reactions, especially in urticaria, H_2_-antagonists have only been documented to reduce wheal, flare, and itching sensation during skin prick test (SPT) procedures [8] and to improve cutaneous symptoms in small studies when administered in conjunction with H_1_-antagonists [9–11]. In an early study evaluating the use of anti-histamines in a mouse model of peanut-induced anaphylaxis, the co-administration of an H_1_- and H_2_-antagonist failed to demonstrate any improvement in the severity nor course of the anaphylactic reaction [12]. Findings from a recent retrospective study in humans have demonstrated that, when used alone, H_2_-antagonists appear unable to inhibit reactions associated with a histamine control during a SPT. Only when administered in conjunction with additional medications possessing anti-histamine properties the H_2_-antagonists demonstrate increased efficacy in suppression of histamine control-induced SPT reactions, likely due to the effects of the additional medications [13]. Research by Fedorowicz et al. are in agreement with these findings, concluding that the evidence supporting the use of H_2_-antagonists in the treatment of urticaria is weak and unreliable [14]. In addition, a small randomized, double-blind, placebo-controlled study suggested that the combination of cetirizine and ranitidine was not more effective than cetirizine alone in the treatment of chronic urticaria [15]. These findings are in contrast to a recent study specifically evaluating cholinergic urticaria suggesting that addition of an H_2_-antagonist (e.g. lafutidine) to ongoing H_1_-antagonist therapy could significantly reduce both objective and subjective symptoms and improve the patient’s quality of life [16]. This finding agrees with a previous retrospective cohort study that evaluated the efficacy and usefulness of adding lafutidine as an adjunct therapy in patients with idiopathic chronic urticaria that was not sufficiently controlled by an H_1_-antagonist alone. 74 % of patients considered lafutidine as useful or better after 3 months of treatment [17]. However, it should be noted that cholinergic urticaria is not solely an IgE-mediated disease, but can involve a complex pathogenesis including cholinergic stimuli, histamine-release, type-I hypersensitivity reactions to sweat and Malassezia globose, and poral occlusion [18].

Cimetidine, the first H_2_-antagonist introduced into clinical practice, has been shown to reduce the suppressor activity of Foxp3+ CD4+ CD25+ Treg cells through an increased degradation of Foxp3 [19]. Additional research has linked the use of cimetidine to an increase in Th1-type cytokine-mediated immune responses and delayed-type hypersensitivity reactions [20]. In a study involving mice sensitized with a ovalbumin, the administration of cimetidine resulted in a significant increase in both specific-IgE and IL-5 levels in the culture supernatants of spleen cells, whereas Th1-, Th2-, and Th17-type cytokines did not differ between treated and untreated mice [21]. Similar results were found in a mouse model of allergic rhinitis in which the mice were sensitized to ovalbumin: the group treated with ranitidine, an H_2_-antagonist, in conjunction with ovalbumin immunotherapy demonstrated higher levels of specific-IgE and IL-13 in their nasal lavage fluid, as well as greater levels of eosinophils in their nasal mucosa tissue, when compared to the untreated control group, suggesting a pro-inflammatory effect of the H_2_-antagonist on specific immunotherapy [22]. In studies of humans, cimetidine has demonstrated additional pro-inflammatory effects including the increased release of Th1-type cytokines, in particular IL-12 and IL-2 [23–25]. Pastorello et al. evaluated sixty-seven patients with systemic allergic reactions to amoxicillin, ranging from mild to severe, and, surprisingly, they found that treatment with an H_2_-antagonist was significantly related to severe reactions (p = 0.007) [26]. In monocytes and dendritic cells, the administration of ranitidine was associated with a reduction in histamine-associated suppression of IL-12 release via the H_2_ receptor [7, 27]. In addition to monocytes and dendritic cells, H_2_-receptor effects were evaluated in basophils collected from patients undergoing venom immunotherapy. Interestingly, the authors detected a rapid upregulation of H_2_-receptor and strong suppression of FcεRI-induced basophil activation and mediator release, including histamine and sulfidoleukotrienes, within the first 6 h of the build-up phase [28].

The majority of these findings appear to suggest that H_2_-antagonists may illicit pro-inflammatory effects, or effects that work in opposition to allergen immunotherapy; however, in 2016, Lee et al. demonstrated that roxatidine presents anti-allergic and tolerogenic effects through the inhibition of NF-κB, a transcription factor involved in immune regulation, apoptosis, cell differentiation, inflammation, and cancer [29]. In particular, roxatidine was able to suppress the release of inflammatory cytokines such as TNF-α, IL-6, and IL-1β from mast cells and delay the fatality rate in anaphylactic-induced mice. Moreover, the authors found that roxatidine significantly reduced ear swelling, mast cell accumulation, cytokine levels, and dendritic cell migration in sections of ear tissue in an animal model of allergen-induced contact hypersensitivity [30]. Additional findings by Geng et al. further support the anti-inflammatory effects of H_2_-antagonists, demonstrating that both systemic corticosteroids and H_2_-antagonists are associated with a significantly greater odds ratio for a negative outcome to the histamine control during SPT [31].

As summarized in Table 1, the scientific evidence supporting the role of H2-antagonists in the treatment of allergic reaction remains conflicted. It is possible that H_2_-antagonists may exert their effects more rapidly on H_2_-receptors located in vessels and on smooth muscles compared to those on immune-cells [16]. Alternatively, when administered as an adjunct to an H_1_-antagonist, it is feasible that they could increase the circulating blood levels via interference in drug metabolism and clearance [32]. Further investigations into the use of H_2_-antagonists during ongoing immunotherapy are needed to evaluate their use in the treatment or prevention of IgE-mediated allergic reactions.

**Table 1:** Summary of the research manuscripts assessing the role of H2-antagonist in IgE-mediated type I hypersensitivity reactions

Article	Animal/human Study	Molecule analyzed	Other therapy (adjunct)	Results
Kupczyk et al. [8]	In vivo human study	Ranitidine		Ranitidine was able to suppress the wheal, flare, and itching sensation in SPT
Runge et al. [9]	In vivo human study	Cimetidine	Diphenhydramine	In acute urticaria, cimetidine + diphenhydramine is more effective than diphenhydramine alone.
Lin et al. [10]	In vivo human study	Ranitidine	Diphenhydramine	The addition of ranitidine to diphenhydramine results in improvement of cutaneous manifestations in patients with acute allergic syndromes
Dhanya et al. [11]	In vivo human study	Ranitidine	Levocetirizine	Levocetirizine + ranitidine resulted in significant reduction of wheal size at 2, 3, 6, and 24 h vs. levocetirizine alone.
Arias et al. [12]	In vivo mouse study	Cimetidine	Mepyramine	The co-administration of H_1_- and H_2_-antagonist had no impact on the severity or the course of anaphylactic reaction
Shah et al. [13]	In vivo human study	Famotidine Ranitidine Cimetidine		H_2_-antagonists had no effect on the positive histamine skin test; if associated with other potentially antihistaminic medications, the odds of a negative histamine control increased. Authors concluded that a 0–2-day discontinuation before testing is recommended.
Fedorowicz et al. [14]	Review on human studies	Famotidine Ranitidine Cimetidine		Evidence for the effectiveness of H_2_-antagonist in urticaria is limited, weak and unreliable. Based on the review, there is not enough evidence to answer the question of whether H_1_- + H_2_-antagonists are better than just H_1_- antagonists alone.
Guevara-Gutierrez et al. [15]	In vivo human study	Ranitidine	Cetirizine	Combination therapy with cetirizine and ranitidine was not more effective than cetirizine alone in chronic urticaria.
Hatakeyama et al. [16]	In vivo human study	Lafutidine	Cetirizine Fexofenadine Bepotastine Ebastine Olopatadine Levocetirizine	Authors concluded that lafutidine can be recommended as an adjunct therapy that improved disease activity and QoL in patients with refractory cholinergic urticaria.
Ogawa et al. [17]	In vivo human study	Lafutidine	H_1_-antagonists (not specified)	In idiopathic chronic urticaria, lafutidine as adjuvant therapy showed a moderate improvement or better in 85 and 76 % of patients after 1– 3 weeks and after 3 months, respectively. Lafutidine was rated as useful or better in 74 % of evaluated patients after 3 months of treatment.
Zhang et al. [19]	In vitro human study	Cimetidine		Cimetidine suppresses the function of Treg cells through a reduction of Foxp3 via E3 ligase Stub1-mediated proteosomal degradation.
Avella et al. [20]	In vivo human study	Cimetidine		Cimetidine therapy prevented a natural decline in delayed hypersensitivity skin tests to four common antigens and significantly increased delayed hypersensitivity, measured by the degree of erythema at both 24 and 48 h and induration at 48 h.
Arae et al. [21]	In vivo, in vitro mouse model	Cimetidine		Administration of cimetidine to Ovalbumin-sensitized BALB/c mice increased serum level of Ovalbumin-specific IgE, IgG_1_ and IgG_2a_. In vitro analysis showed an increased IL-5 secretion by Ovalbumin-stimulated spleen cells.
Shin et al. [22]	In vivo mouse model	Ranitidine		Treatment with ovalbumin immunotherapy + ranitidine showed a significant increase in serum specific IgE levels, nasal lavage fluid IL-13 levels and the number of tissue eosinophils when compared both with immunotherapy alone and with immunotherapy + H_2_-agonist.
Ishikura et al. [23]	In vivo human study	Cimetidine		Cimetidine significantly increases serum IL-12 levels in patient admitted to the intensive care unit
Hahm et al. [24]	In vitro human study	Cimetidine Ranitidine Famotidine		In peripheral blood mononuclear cells from patients with gastric cancer, cimetidine increases the cytotoxicity and proliferative response of lymphocyte to mitogen
Jafarzadeh et al. [25]	In vivo mouse model	Cimetidine		In a mouse model, cimetidine significantly increased both serum levels of IL-2, IL-10, IL-12, and IL-17 and delayed type hypersensitivity responses that are normally suppressed after a burn injury.
Pastorello et al. [26]	In vivo human study	H_2_-antagonists (not specified)		Therapy with H_2_-antagonists was significantly associated with an increase in the risk of a severe reaction to amoxicillin. Patients that had received H_2_-antagonists presented higher levels of specific IgE.
van der Pouw Kraan et al. [27]	In vitro human study	Ranitidine		In human monocytes, ranitidine reversed the inhibition of IL-12 production caused by histamine.
Lee et al. [30]	In vivo, in vitro, human and mouse model	Roxatidine		Roxatidine suppressed both the expression of TNF-α, IL-6, and IL-1β, and the activation of caspase-1, in stimulated human mast cells and in anaphylactic mouse model. In animal model of allergen-induced contact hypersensitivity, roxatidine significantly reduced ear swelling, mast cell accumulation, cytokine levels, and dendritic cell migration in sections of ear tissue.
Geng et al [31]	In vivo human study	H_2_-antagonists (not specified)		The administration of H_2_-antagonists is associated with a significant odds ratio for a negative histamine response at prick test.
Simons et al. [32]	In vivo human study	Cimetidine	Hydroxyzine	Hydroxyzine + cimetidine showed a significant increase both in serum hydroxyzine concentrations and in suppression of the histamine-induced wheal and flare.

## Data Availability

Not applicable.

## Abbreviations

SPT: Skin prick test
